# Editorial: Physiological response to environmental stressors in invertebrates

**DOI:** 10.3389/fphys.2022.1002192

**Published:** 2022-09-30

**Authors:** Zbigniew Adamski, Nikoletta Ntalli, Małgorzata Słocińska, Laura Scrano

**Affiliations:** ^1^ Department of Animal Physiology and Developmental Biology, Faculty of Biology, Adam Mickiewicz University in Poznań, Poznań, Poland; ^2^ Laboratory of Electron and Confocal Microscopy, Faculty of Biology, Adam Mickiewicz University in Poznań, Poznań, Poland; ^3^ School of Agricultural Sciences, University of Thessaly, Volos, Greece; ^4^ Department of European and Mediterranean Cultures, University of Basilicata, Matera, Italy

**Keywords:** homeostasis, invertebrate, environmental stress, environmental pollution, climate change, global warming

The growing pressure of anthropogenic activities on ecosystems and the ongoing climate change, as well as natural phenomena, influence the set of organisms organized in communities and ecosystems. Among the stressors that affect organisms are (among others): global warming and the resultant increase of temperatures of water and terrestrial habitats, the spread of pollutants e.g., metals, pesticides, persistent organic pollutants, or drugs. All organisms, subjected to the influence of these agents, react in altered metabolism, malfunctions of physiological processes, or behavioral alterations. If not balanced, these changes may lead to significant changes in populations and whole ecosystems. Importantly, the human-derived negative stimuli are often sudden, which disturbs the homeostasis in organisms.

**FIGURE 1:**
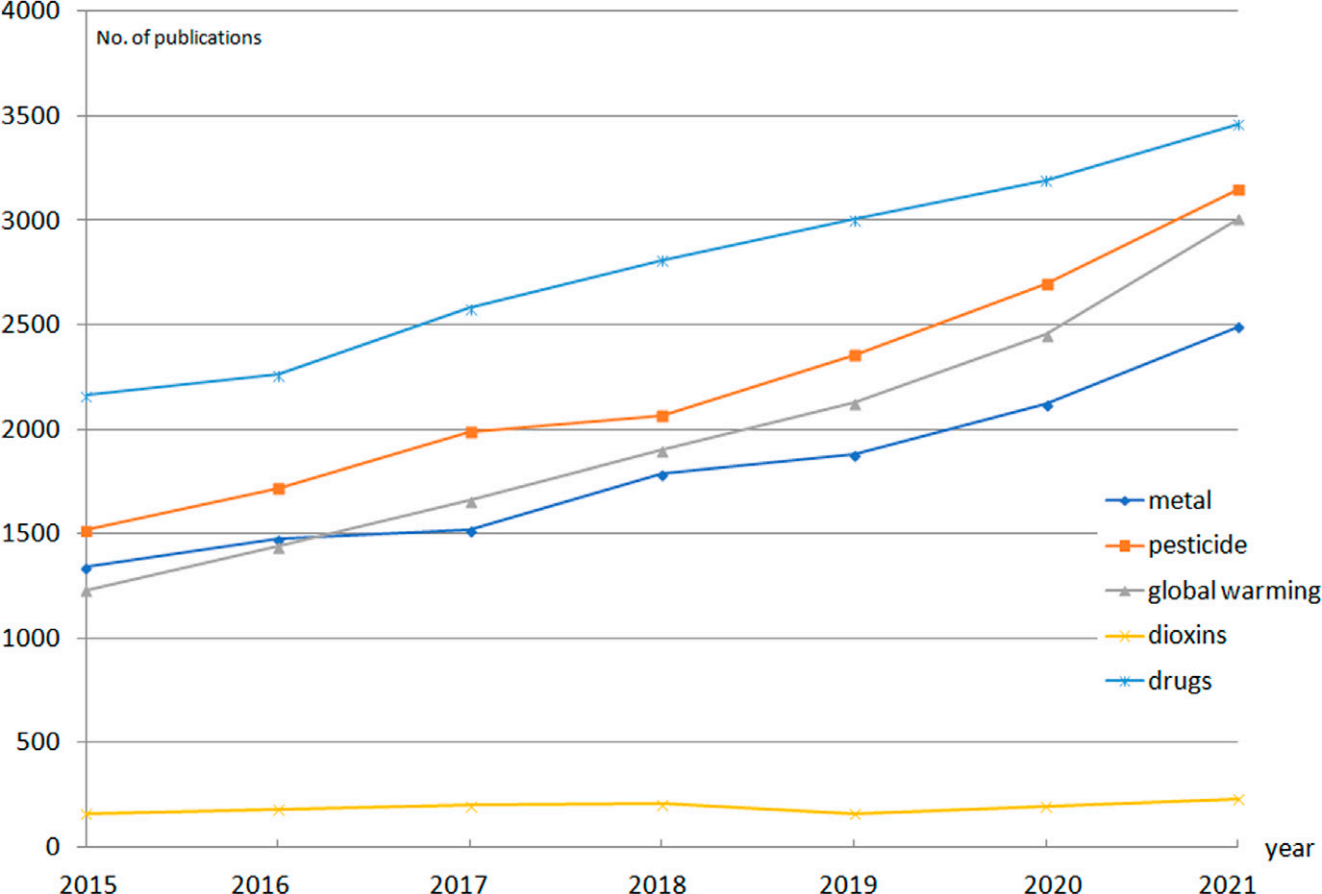
The number of papers published in scientific journals, dealing with the selected anthropogenic stressors and invertebrates. Based on results from Scopus search: a stressor + environment + invertebrate (as of 22 June 2022)

The importance of responding to the growing anthropo-pressure has repercussions in scientific literature. There is a growing interest of the scientific community in the effects of anthropogenic activity on living organisms. A brief analysis of the number of papers published on this topic shows the increasing trend of publications concerning pollutants, the environment, and invertebrates (Figure 1). These animals are used as models in life sciences and bring important data concerning response to environmental conditions (Sun et al., 2021; Lubawy et al., 2022). For this reason, we proposed the research topic concerning the answer of invertebrates to anthropogenic stressors. In this frame we discuss the wide range of physiological responses, given by invertebrates, as a response to environmental stressors. Indeed, the published manuscripts indicate a range of stressors, like salinity, temperature, CO_2_-acidification and desiccation. The papers deal with the important groups of invertebrates: insects and crustaceans. Also, the range of the levels of biological organization, tested within the studies was wide: a transcription factor p53, heat shock proteins (Hsp70 and Hsp90), enzymatic activity, cellular studies, metabolism, reproduction, locomotion, morphology, and survival. As one can notice, the authors of the present Research Topic focused on the global-change threats. Among the most important findings, we may find the results indicating the importance of p53 proteins, which take part in the regulation of cell cycle, for the survival in the altered salinity of water as a habitat, significance of Hsp proteins in response to the environmental stress, relation between altered physical properties (water salinity, acidification, and desiccation) and development of representatives of invertebrates.

The presented results show the complicity of the effects of stressors, as well as the importance of keeping homeostasis at all levels of biological organization. Ren et al for the first time reported that the expression of p53 mRNA and p53 protein levels are increased in case of altered salinity. Moreover, apoptosis and activity of antioxidant enzymes in the gills of arthropods exposed to stress varied significantly. The prooxidant-antioxidant balance is crucial for the homeostasis of organisms, and the imbalance may lead to numerous important diseases within organisms and play an important role in various pathological conditions, leading to cell damage. Next, Ding et al. (2021) presented an interesting study proposing the management, biological prevention, and control of a lepidopteran pest, *Glyphodes pyloalis*, based on studies of Hsp proteins. They also highlighted these proteins’ role in response to stressors. On the other hand, Leiva et al. presented a set of data showing the classical toxic effects: survival, biomass, development, and morphology. Interestingly, they integrated the abovementioned effects with the metabolic rate and antioxidant-enzyme activity. Their results clearly show the thread caused by anthropogenic changes in the biosphere of living organisms, including endangered species, and a wide range of levels of biological systems. Engell Dahl and Renault showed the effects of desiccation–which may coincide with the effect of global warming–on a beetle, *Alphitobius diaperinus*. They also focused on the similar effects on the level of organisms: survival, locomotion, and reproduction. The tenebrionid beetles are quite resistant to the lack of water, as they often live in crop and food stores. However, the stress led to reduced fertility. Together with the previously mentioned research of Leiva et al. (2022), both results show the possible “secondary” consequence of global changes—relatively low effects on pests, with simultaneous detrimental effects on endangered, or economically important species.

Taken together, the papers presented in this research topic show complexity of effects caused by environmental stressors on invertebrates. The imbalance of homeostasis can be observed at all levels of biological organization, from biomolecules, through suborganismal levels, up to the levels of individual organisms and populations. This, in consequence, may lead to drastic changes within ecosystems. The results indicate the importance of multi-level research on the effects of stressors on invertebrates.
